# The P-factor and its genomic and neural equivalents: an integrated perspective

**DOI:** 10.1038/s41380-021-01031-2

**Published:** 2021-02-01

**Authors:** Emma Sprooten, Barbara Franke, Corina U. Greven

**Affiliations:** 1 Departments of Cognitive Neuroscience and Human Genetics, Donders Institute for Brain, Cognition and Behaviour, Radboud University Medical Center, 6525 EN Nijmegen, The Netherlands; 2 Departments of Human Genetics and Psychiatry, Donders Institute for Brain, Cognition and Behaviour, Radboud University Medical Center, Nijmegen, The Netherlands; 3 Karakter Child and Adolescent Psychiatry University Center, 6525 GC, Nijmegen, The Netherlands; 4 Social, Genetic & Developmental Psychiatry Centre, Institute of Psychiatry, Psychology & Neuroscience, King’s College London, London, SE5 8AF UK

**Keywords:** Neuroscience, Psychiatric disorders, Genetics

## Abstract

Different psychiatric disorders and symptoms are highly correlated in the general population. A general psychopathology factor (or “P-factor”) has been proposed to efficiently describe this covariance of psychopathology. Recently, genetic and neuroimaging studies also derived general dimensions that reflect densely correlated genomic and neural effects on behaviour and psychopathology. While these three types of general dimensions show striking parallels, it is unknown how they are conceptually related. Here, we provide an overview of these three general dimensions, and suggest a unified interpretation of their nature and underlying mechanisms. We propose that the general dimensions reflect, in part, a combination of heritable ‘environmental’ factors, driven by a dense web of gene-environment correlations. This perspective calls for an update of the traditional endophenotype framework, and encourages methodological innovations to improve models of gene-brain-environment relationships in all their complexity. We propose concrete approaches, which by taking advantage of the richness of current large databases will help to better disentangle the complex nature of causal factors underlying psychopathology.

## Introduction

A trend has emerged that shows striking parallels between clinical psychology, psychiatric genetics and neuroimaging. The dense covariation between a range of psychopathology-related traits is described in terms of new constructs. These take the form of three general dimensions or factors: a general psychopathology factor or “P-factor” [1, 2], a general dimension of genetic liability for psychopathology [3, 4], and a general dimension of brain structure and function [5, 6]. Here we refer to the general dimension derived from behavioural data as “phenotypic P-factor”, the general dimension derived from genomic data as “genomic P-factor”, and general dimensions derived from neuroimaging as “neural P-factors”. Whilst these general dimensions are receiving ample attention within each discipline, little has been done to interpret them together.

This Perspective Article has three main aims: (1) to integrate knowledge from the three types of P-factors into a new unified theory; (2) to evaluate implications of this theory for widely held assumptions behind endophenotypes; (3) to translate the theory into concrete future directions for psychiatric genetics and imaging genetics. In the first section, we introduce each of the general dimensions individually. This section is on purpose brief and not meant as an exhaustive literature review. The second section is the core of this paper, and where its novelty lies. There, we advance previous interpretations of genetic and phenotypic P-factors [7, 8] by integrating the neural P-factor into a unified theory of all three P-factors (aim 1), and by discussing important implications of this theory for the endophenotype concept in psychiatry (aim 2). In the third section, we translate our new theoretical perspective on gene-brain-behaviour interplay into concrete strategies for future research (aim 3).

## General dimensions describing inter-individual variation in psychopathology, genetic liability for psychopathology, and brain structure and function

### A phenotypic P-factor of psychopathology

Psychiatric disorders have fuzzy boundaries in their clinical definitions and phenomenological presentations. Few symptoms are unique to any diagnosis. A symptom can be a contributing criterion to multiple disorders, and a common comorbidity across disorders. Moreover, disorders may develop into other disorders through maturation and ageing (i.e., “network theory”) [8–10]. Using structural equation modelling (SEM), several studies have identified a single common psychopathology dimension, or “P-factor” [1, 2, 11–16], which captures a significant portion of inter-individual variation (e.g., 23% [16]) in the presence and severity of psychiatric symptoms in the population. This phenotypic P-factor is a reflection of the correlation structure across symptom scales and the comorbidity of psychiatric disorders [8]. Whilst further interpretation of the P-factor itself has already received much attention in the clinical and behavioural domain [8, 17–19], its relationship to emerging general constructs in genetics and neuroimaging has not been addressed in detail.

### A genomic P-factor

The phenotypic P-factor is heritable [20–22], and phenotypic overlap may be partly driven by shared genetic liability [12]. Twin and family studies have long shown that genetic risk for psychiatric disorders is indeed shared across diagnostic categories [23, 24]. More recently, this shared genetic risk has also been observed from single nucleotide polymorphisms (SNPs) in DNA [25–27]. For example, attention-deficit/hyperactivity disorder (ADHD), anxiety disorders, major depressive disorder, bipolar disorder and schizophrenia all have pairwise genetic correlations exceeding ~0.20, ranging up to ~0.75 [26].

To identify a genomic dimension that captures this shared genetic liability, SNP-based genetic correlations have been further analysed using genomic SEM [3] and principal component analysis (PCA) [4]. A single genomic factor (or “polygenic P-factor” [4]) containing genome-wide factor-loadings representing each SNP’s contribution to cross-disorder liability, was derived. This genomic P-factor fitted well to the data [3], explained 20%-43% of the SNP-effects across disorders [4], and improved power for genome-wide association study (GWAS) [3].

### A neural P-factor

The phenotypic P-factor is associated with brain structure and function [28–36]. A meta-analysis of structural magnetic resonance imaging (MRI) studies showed that case-control differences of six psychiatric disorders co-localized to the same brain regions [37], while very few diagnosis-specific associations were seen. Recent meta-analyses from the ENIGMA Consortium confirm this neuroanatomical overlap across adult psychiatric disorders [38, 39], but less so for neurodevelopmental disorders [39, 40]. Regarding functional MRI, a meta-analysis of case-control studies disorders also revealed highly significant spatial overlap across five psychiatric disorders, in the absence of diagnosis-specific effects [41].

Akin to the phenotypic and the genetic domain, these cross-diagnostic similarities have led to the construction of general dimensions describing inter-individual variation in neuroimaging traits. Such “neural P-factors” have been identified from functional connectivity [6], diffusion MRI [42], neuroanatomical data [5], and multi-modal data encompassing all the above [43]. In white matter, a single whole-brain factor of diffusion anisotropy was associated with the phenotypic P-factor in a community sample of 10-year old children [42]. In the resting-state connectivity study, a single dimension capturing a combination of functional connectivity, with environmental and behavioural variables was identified, which explained up to 17% of variance in input variables. The traits in this combined phenotypic/neural P-factor showed an interesting pattern with traits generally regarded as positive (intelligence, life satisfaction, educational attainment) at one end of the spectrum, and traits generally regarded as negative and associated with psychiatric liability (substance abuse, poor sleep quality, aggression, stress) on the other end [6]. An analogous neural P-factor based on structural MRI was recently described in the same sample, and correlated with the same behavioural, demographic and environmental measures [5]. A similar multimodal neural P-factor explained 10–40% of variance in environmental and demographic variables in an independent sample of children and adolescents [43].

In summary, recent neuroimaging and genetic research across psychiatric traits mirrors observations at the phenotypic level. Psychiatric symptoms and their biological correlates co-vary highly in the population. Within each discipline, this covariation can be captured by single phenotypic, genomic and neural P-factors.

## An integrative interpretation of the general dimensions

### Explaining variance versus understanding mechanisms

Together, the scientific developments described above mark a perhaps unsurprising, yet new development in our understanding of biological underpinnings of psychiatric disorders. Decades of work on pairwise associations between genome, brain, and single diagnoses generally revealed only modest effects. In comparison, the diagnosis-general phenotypic, genomic and neural P-factors explain undeniably large amounts of inter-individual variation. Whilst this quality is immediately appealing from a statistical perspective, it does not directly lead to mechanistic insights or clinical impact [44]. As the P-factors are built solely from covariance structures, they are arguably ultimate cases of “correlation without causation.” Yet, we know from basic and experimental neuroscience that the associations between the genome, brain and behaviour are governed by tightly regulated mechanisms of molecular pathways and protein interactions. To arrive at a new integrated perspective on potential mechanisms underlying the different P-factors, we first discuss some technical and conceptual differences between the three P-factors.

### Methodological differences between the P-factors

Tables 1 and 2 summarize key methods used to extract P-factors in each discipline. Generally, the phenotypic P-factor has been derived from continuous symptom ratings in population-based samples [1, 2, 14]. Whilst different designs and approaches have led to inconsistent results for specific cross-diagnostic factors, such as “thought disorder” or “fear and distress” factors, the general P-factor has been largely consistent, regardless of demographics or diagnostic instrument [1, 2, 12–14, 17]. The finding that binary diagnostic phenotypes and continuous dimensional traits yield similar results is in line with taxometric properties of phenotypic variability in the population [45] and the “liability threshold model”, under which diagnoses are the extremes of latent continuous traits [46].

**Table 1:** Glossary.

Genetics	
Heritability	The proportion of variance of a phenotype that is attributable to genetic factors.
Genetic correlation	The degree to which two phenotypes are influenced by the same genetic variation.
GWAS	Genome-wide association study: mass-univariate analysis to relate common variation over the entire length of the DNA to a phenotype of interest.
SNP	Single nucleotide polymorphism: a (common) genetic variation in the DNA sequence where different alleles (nucleotides) can exist in the population.
Polygenic	Influenced by many genetic variants (i.e., hundreds, or thousands of genes), as opposed to monogenic (influenced by a single gene, or single genetic variant).
Mendelian Randomisation	Hypothesis-driven method aimed at inferring causality from (cross-sectional) associations between a genetic variant and two or more phenotypes. E.g. to test whether a modifiable behavioural or neural trait potentially mediates the effect of a genetic variant on a disease [95].
LD-score regression	Linkage-disequilibrium score regression: method to calculate genetic correlations on the basis of GWAS output (i.e., “summary statistics”), given the relationship of the statistics to each variant’s linkage disequilibrium pattern [25]
Neuroimaging	
MRI	Magnetic Resonance Imaging
Functional MRI	MRI acquisition method to estimate regional brain activation based on local blood-oxygen level dependent (BOLD) signal.
Diffusion MRI	MRI acquisition method to measure microstructural tissue properties based on direction and amount of diffusion of water molecules. Most often used for investigating white matter fibres.
Functional connectivity	The degree to which two or more brain regions show similar activation patterns over time, based on the correlation or mutual dependence of their BOLD time-series.
Multivariate methods	
PCA	Principal Component Analysis: data-driven data reduction method to extract maximally uncorrelated components (i.e., “factors”) from many variables.
ICA	Independent Component Analysis: data-driven data reduction method and source identification method, which extracts maximally independent components (i.e., “factors”) from many variables.
CCA	Canonical Correlation Analysis: method to extract modes (here:“factors”) across two or more sets of variables (e.g., MRI and behavioural variables), such that the variables within a mode are maximally correlated.
SEM	Structural Equation Modelling: data reduction method to fit a priori factor structures to data and extract these factors. Can be confirmatory (1 model is tested) or exploratory (multiple a priori models are tested and compared).

^*^Note: For consistency and clarity, throughout the paper the term “factors” is used to describe all kinds of factors, components, dimensions, sources, or modes, even if the term “factors” is unusual for the particular method that was used. For the purpose of the present paper, the interpretation is the same across these terms.

**Table 2:** Summary of the 3 P-factors and the methods by which they were derived.

	Statistical methods with relevant information	Design	Psychopathology Assessments	Key references [1]
Phenotypic P-factor	Most common methods: SEM, factor analysis These are variance decomposition methods and path diagrams within the phenotypic domain. SEM and factor analysis are generally exploratory of multiple a priori models and/or a test of predefined factor structure	• Generally population based • High-risk cohort [13]	• Generally continuous symptom ratings • Binary diagnosis [12] • Probability of diagnosis [13]	[1, 2, 11–16]
Genomic P-factor	Most common methods: PCA, Genomic SEM These are variance decomposition methods on the genetic correlations across disorders, which are derived using LDSR of GWAS stats [3], SNP-based methods [4], or family/twin-based approaches. PCA is fully data-driven, while Genomic SEM is exploratory or confirmatory of a hypothesized factor structure.	Case-control GWAS designs	Clinical diagnosis	[3, 4, 27, 102]
Neural P-factor	Most common methods: CCA, ICA CCA can be used to extract “modes” across behavioural/environmental and MRI measures simultaneously. A mode is driven by a combination of the correlations within and across the variable classes. ICA tends to be run within the MRI domain. The resultant “components” can then be correlated with the behavioural and environmental variables. Both ICA and CCA are fully data-driven and are useful to extract a large number of “factors” from high-dimensional data.	• Population based, adults (Human Connectome Project) • Children and adolescent population, enriched for mental distress (ABCD Cohort)	• Behavioural, demographic & environmental measures • Self-report of (family) diagnosis and substance use • Cognitive task performance	[5, 6, 39, 43]

Note: For consistency and clarity, throughout the paper the term “factors” is used to describe all kinds of factors, components, dimensions, sources, or modes, even if the term “factors” is unusual for the particular method that was used. For the purpose of the present paper, the interpretation is the same across these terms.

^1^this references list is not exhaustive.

Similar to the phenotypic P-factor, neural P-factors tend to be derived from continuous traits in population-based cohorts [5, 6, 42]. In contrast, genomic P-factors are generally based on case-control comparisons [3, 4]. The degree to which the clinician-based case-control genomic P-factor can be generalised to population-based traits has been questioned [47]. This design-specific effect may be particularly strong for psychotic disorders, whereas case-control GWAS of depression [48], ADHD [49], and autism spectrum disorders [50, 51] show high overlap with continuous traits in community samples.

We ought to keep in mind these methodological differences until this has been tested directly (see Section 3). Nevertheless, we hypothesise that the three types of P-factors reflect to some degree the same inter-individual variation, on the basis of their similarities.

### Similarities between the P-factors

The three P-factors share several key properties:

1. They are based on the high degree of covariance between many variables.
2. They describe substantial amounts of inter-individual variation.
3. They are diffuse, comprised of subtle, widespread effects throughout the phenotypic, genomic, and neural domains, as opposed to restricted to a few brain regions, genomic loci, or behavioural domains.
4. They do not infer directions of causality (except for DNA, which due to its stability is more likely cause than effect).
5. They are associated with heritable variables that are rather “environmental” in nature. The phenotypic P-factor and the neural P-factors are strongly associated with household income, years of education, and welfare benefit use [1, 5, 6]. While the genomic P-factor has not yet been directly associated with environmental variables, many environmental factors are genetically correlated with psychiatric disorders [7, 52, 53].

The shared properties 1-3 and 5 suggest that the three types of P-factors, despite their differences, explain at least some similar aspects of inter-individual variation. The 4th shared property—the lack of causal information—is a critical limitation that impedes translation from correlations to mechanisms, and ultimately to clinical impact. We propose that the 5th property—the association with the heritable environment—may be particularly important in disentangling these potential causal mechanisms.

### The heritable environment and gene-environment correlations

In the context of quantitative genetics, the “heritable environment” may seem paradoxical, because in genetics “environment” tends to equal everything that is not explained by genetic variation. Here, we mean by “environment” the common sense conceptual notion, i.e., variables mainly located outside the body. Environmental variables like recreation, educational attainment and socio-economic status have heritability estimates around 40–50% in twin studies and 5–27% in SNP-based estimation [52, 54–58].

The heritability of environmental measures revives seminal papers of Plomin et al. [59], Kendler et al. [60], and Scarr and McCartney [61], who first described the importance of possible gene-environment correlations (rGE) in this context. RGE occurs when exposure to an environmental risk factor is influenced by the same genetic variation as a (psychiatric) trait of interest [59, 62]. As a consequence, rGE can give rise to “environmentally *mediated* pleiotropy” [7, 63, 64]. For example, hypothetically genetic variation influencing one’s degree of openness to new experiences in turn may influence a person’s attitude toward substance use, which is a risk factor for several psychiatric disorders. Although scarcely considered in the GWAS era so far [62], rGE is abundant in psychiatric genetics. For example, household income and educational attainment are genetically correlated with multiple psychiatric disorders [52, 53]. In line with the possibility of “environmentally mediated pleiotropy” [4, 7, 63], statistically removing the variance of socio-economic status significantly alters genetic correlations between psychiatric disorders [65].

Importantly, many of these environmental variables also have spatially overlapping associations with brain traits [5, 6, 43], which is captured by the neural P-factor (see section 1.3). Therefore, the consideration of gene-environment correlations has important consequences for the identification of brain mechanisms on the causal pathway from genes to behaviour.

### Reconsidering the brain as mediator between genes and behaviour

**Fig. 1:**
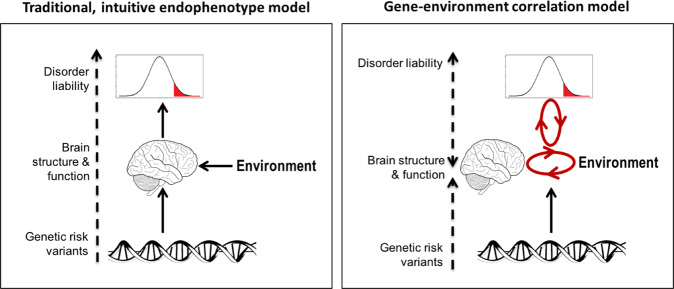
The brain is mediator in theory, but not necessarily in practice. Intuitive theories and concepts, like endophenotypes, logically assume that genetic effects on behaviour (and on environment) pass through the brain. However, the heritable environment induces rGEs, which propagate over time, through brain, environment and behaviour. Hence, the model is updated with a causal brain-behaviour-environment loop. This loop permits a multitude of causal mechanisms between the genome and behaviour, inside and outside the brain, and we propose that this is reflected in the cross-trait covariance captured by the P-factor and its genetic and neural equivalents. Note that the exact mechanisms by which the environment influences the brain and behaviour are many, and may potentially involve tissues outside the brain. For the sake of clarity, we here do not include routes from genome to behaviour that are outside of the brain.

Brain structure and function are heritable [64, 66–69], and theoretically, the brain is the mediator in the causal chain from genetic effect to behaviour and environment. Therefore, studying brain traits - as “endophenotypes” - in relation to genetic risk factors is generally considered to help unravelling causal mechanisms from genome to behaviour [70–73]. However, the complexity of environmentally mediated pleiotropy [4, 7, 63] also holds for genetic influences on the brain, and challenges the bottom-up causality from genome to brain to behaviour (Fig. 1). Consequently, the causal chain from genes to behaviour includes a brain-environment loop. For example, the SNPs that confer risk for major depression may influence the brain directly throughout development, but many of these SNPs also contribute to environmental exposures such as household income [52], social deprivation [52], traumatic experiences [74], poor sleep [75], socio-economic status [76], to name a few. Some of these factors will affect the brain as well. These indirect, environmentally mediated effects accumulate over time. Thus, in theory, the brain is the mediator between the genome and behaviour, but in practice the genetic effects on the brain we measure can be anywhere on the causal brain-environment loop that perpetuates and broadens from the moment of conception to the moment of MRI measurement. Considering the number of causal routes to psychopathology this permits, it should be no surprise that psychiatric disorders and their associated brain traits are highly polygenic [77–79], and non-specific to brain regions, tissues or circuits [50, 80, 81]. In line with Avinun et al. (2020) [7], we argue that this convolution of many causal routes induces not only highly correlated and subtle effects in the genome, but also in the brain, and these are now captured by general dimensions in the form of P-factors.

### Summary: an integrated interpretation of three P-factors

To summarise, the phenotypic, genomic and neural P-factors all capture inter-individual variation extremely efficiently in a single variable. However, this efficiency does not readily translate to mechanistic insights. We note an important contribution of heritable environmental risk factors as a common feature to all three P-factors. Insofar as we can speculate on causal relationships, it seems likely that variables within and across dimensions have bidirectional causal relationships, at least when considered throughout the human lifespan. In this context, we emphasise that heritable environmental risk factors can, just like neural processes, be mediators on the causal pathway from genes to behaviour. Thus, as a general conclusion, general dimensions of psychopathology and genetic liability for psychopathology reflect a dense web of mutually reinforcing traits that propagate throughout development, via the environment and the brain, in conferring risk for psychiatric disorders. While the P-factors in the brain and the genome may not be the only explanation for all patterns at the phenotypic level, our interpretation also fits with “network theory”:[9, 10] the web of mutually reinforcing traits may, at different stages of development and under different circumstances, clinically manifest as a sequence of different symptoms or diagnoses.

## Onwards: quantifying and using gene-environment correlations

Our conclusion may appear daunting: if all risk factors are correlated and their causal effects are bidirectional, it is difficult to dissect their relationships into mechanistic insights that can be therapeutically interfered with. However, it is only by acknowledging this complexity that we can make headway in biological psychiatry, for example using imaging genetics. Both neuroimaging and genetics studies have been troubled by lack of replication and limited clinical impact. This is largely due to subtle and widespread effects in the genome and the brain, which call for new approaches that can more effectively model these patterns of effects. We finally have large sample sizes to work with (e.g. UK Biobank [64, 82], ENIGMA [83, 84], PGC [27]), and a range of new methods to apply to them.

Concretely, we consider the following strategies to move toward understanding biological mechanisms underpinning the P-factors:

1. To test directly to what extent the three P-factors reflect the same inter-individual variation.
2. To quantify rGEs using GWAS data and extract *multiple* dimensions, which may reflect more distinct mechanisms.
3. To extend multivariate genetic models to include data types at multiple molecular and neurophysiological scales of investigation.
4. To apply multivariate models to longitudinal cohorts, to get a glimpse of sensitive age-windows and possibly causal dynamics of gene-brain-environment interplay.

### Testing to what extent the three P-factors reflect the same variation in the population

Different designs and statistical approaches were used for deriving the three P-factors (Table 2 and Section 2.2). Using the same statistical method across phenotypic and brain traits and applying it to the same sample, would be a first step to understand the shared biological mechanisms behind these new constructs and help identify any potential biases and confounders in their derivation. In addition, the validity and generalisability of the P-factors needs to be examined across healthy and clinical populations of different ancestries and demographics (e.g., age, sex).

### Quantifying genetic correlations and gene-environment correlations using GWAS data

Classic twin models can be extended to multivariate models to quantify the shared genetic influence on multiple variables, including environmental measures [85, 86]. This way, rGEs can be quantified. For example, the effect of exposure to aggressive media on aggressive behaviour in children may be partly explained by genetically driven media preferences [87]. Since multivariate models can also be applied to GWAS output [25], their scope and feasibility has increased. For example, using LD-score regression [25], genetic overlap between socio-economic status and several major psychiatric disorders and risk factors was quantified, and accounting for genetic variation of socio-economic status changed the genetic correlations between those psychopathology traits [76]. In addition, multivariate models like genomic SEM can generate not only a single general factor, but also more specific factors [27]. Similarly, at the phenotypic level, two or three more specific factors are often derived [1, 2, 13, 14, 33, 45, 88, 89]. The high dimensionality of neuroimaging data already tends to be described by dozens of dimensions rather than a single one [5, 36, 43]. Combining data more systematically across disciplines and drastically increasing the number of phenotypes to hundreds or more, would allow better estimation of many more genomic factors as well. Table 2 describes data-driven methods suitable for such high-dimensional multivariate analyses. Multiple independent factors may expose more specific patterns of gene-brain-behaviour associations, thereby aiding interpretation in terms of concrete mechanisms. In the genome, for example, bioinformatics [90, 91] may reveal that multiple independent factors map to molecular pathways or cell types with higher sensitivity and specificity than a single general factor. This could ultimately help to disentangle multiple specific mechanisms from the web of correlated risk factors.

### Including multi-level neurobiological data at multiple scales of investigation

We focused mostly on integration of neuroimaging, genomic and phenotypic research. However, the underlying mechanisms involve every step from alleles, through molecular interactions, cell morphology, neural circuits to behaviour [92], and even non-brain related mechanisms that contribute to behaviour. The examples of multivariate models above can be extended with multi-layer biological information, including transcriptomics, epigenetics, and proteomics. Doing so could yield new P-factors and more specific factors (as described above) *across* biological levels of investigation. Just like in genomic SEM, which is based on the covariance of SNP-phenotype associations, covariance matrices of methylation-brain or transcriptome-brain associations be decomposed into novel constructs whose factor loadings indicate which variables across levels work together in influencing phenotypes of interest. For example, N-dimensional PCA of transcriptome-neuroimaging associations returns N factors containing loadings of brain measures, plus N respective vectors of transcript loadings. Transcripts and brain measures loading highly on the same component jointly describe a distinct portion of brain-transcript covariance, and therefore may point to a distinct mechanism of how gene expression influences brain structure and function, or vice versa. Neural and transcript-dimensions can be further linked to molecular pathways [90, 91] and atlases of brain function [93], which while allowing no direct causal inference, help generate new testable hypotheses for experimental studies to test causality. To model many variables with complex relations and different distributions, new methods such as MiXeR [94] could be considered to quantify genetic overlap more flexibly, while Mendelian Randomization [95] could give first glimpses of causal inference.

### Modelling the dynamics of gene-environment correlations over time

Longitudinal twin research indicates that the phenotypic P-factor and its genetic underpinnings are largely stable over time [96]. Extensions of longitudinal genetic models can give insights into which gene-brain-environment associations and interactions are most relevant for psychiatric symptoms at different age-windows, and as a first test of causality. For example, a longitudinal twin study suggests that the genetic influence on temperament at age 3 years partly determines peer problems at a later age [97]. Currently, SNP-based analyses have several advantages over twin studies: GWAS data are currently more widely available than twin samples; and SNP-based results can be further investigated to understand the molecular basis of the general and specific factors. Established methods from twin research provide a ready-made framework for further extending SNP-based approaches [25, 98] to better understand mechanisms underlying rGEs. Although longitudinal modelling of single variables has been performed [99, 100], further extensions of these models including environmental variables and/or multivariate genomic, neural and psychopathology factors reviewed above, are now feasible with the increased availability of summary statistics of large consortia [27, 64, 82, 83, 101].

## General conclusion

Pioneers of P-factor concept wrote: “Correlations [between different symptom dimensions] are not a problem, but a profoundly important source of information about the nature of psychopathology.” [17]. The same is true for the highly correlated biological risk factors of psychopathology we reviewed here. In the last few decades, the limited clinical impact of psychiatric genetics and neuroimaging in psychiatry has been largely attributed to two issues: (1) tiny effect sizes in any specific (genomic or neuroanatomical) location, and (2) a lack of specificity to diagnoses or symptoms. Following our integrated interpretation of the recently identified general dimensions in psychopathology, psychiatric genetics, and neuroimaging, our perspective is that the solution to these problems lies in accepting the complexity of the nature of the causal mechanisms we aim to find, and in modelling them more accurately. We suggest that the neural P-factor, like its genetic and phenotypic equivalents [7], reflects an abundance of rGEs underlying multifactorial neuropsychiatric traits. The expansion of available data and new multivariate methods provide promising new ways to account for and quantify the nature of the covariance of psychopathology with a multitude of biological and environmental risk factors. In the near future, we anticipate exciting new research that will take our understanding of gene-brain-behaviour-environment relationships from a web of associations to new hypotheses of causal relationships.
